# TRANSLATION AND CULTURAL ADAPTATION OF A DEMENTIA EVIDENCE-BASED PROGRAM FOR FAMILY CAREGIVERS

**DOI:** 10.1093/geroni/igae098.0853

**Published:** 2024-12-31

**Authors:** Roxana Delgado, Carole White, Daria Neidre, Kimberly Peacock, Luis Luy

**Affiliations:** The University of Texas Health Science Center San Antonio, San Antonio, Texas, United States; The University of Texas Health San Antonio, San Antonio, Texas, United States; The University of Texas Health San Antonio, San Antonio, Texas, United States; The University of Texas Health San Antonio, San Antonio, Texas, United States; The University of Texas Health San Antonio, San Antonio, Texas, United States

## Abstract

Culture influences how family caregivers provide care. The role of culture is especially critical when assisting care recipients with complex conditions like Alzheimer’s Disease and other related dementias. The “Aprendiendo Juntos/Learning Together” intervention is a cultural adaptation of “Learning Skills Together”, an evidence-based intervention to support Latino family caregivers in providing care for a family member with dementia. Adapting an evidence-based intervention should focus on language translation as well as integrating cultural values and practices. The adaptation of the 6-week virtual intervention “Aprendiendo Juntos/Learning Together” used a community participatory approach to inform the intervention’s structure, content, and design. Guided by an Advisory Council of Latino family caregivers, we used a mixed methods design consisting of surveys and individual interviews with Latino family caregivers (n=13) to inform the intervention adaptation. Three main recommendations for adaptation based on our partnership with the advisory council and the results of the individual interviews related to 1) tailoring of the content around dementia, 2) greater focus on caregiver self-care, and 3) care planning. The adapted intervention was tested using a randomized wait-list control design. We enrolled 46 Latino family caregivers, n=32 English-speaking participants, and n=14 Spanish-speaking participants. Recruiting Latino caregivers through community partners and using snowball sampling amplified the reach to the community. Testing of the adapted intervention has been completed. Given the cultural sensitivity of a group’s unique challenges, barriers, and needs, rigor in the methods is critically important in the adaptation process.

